# Research Progress on Natural Products That Regulate miRNAs in the Treatment of Osteosarcoma

**DOI:** 10.3390/biology14010061

**Published:** 2025-01-13

**Authors:** Lin Wang, Xinyu Liu, Haoze Lv, Han Zhang, Rimei Lin, Shan Xu, Chaojing Zhang, Shilei Lou, Zhidong Qiu, Cong Sun, Ning Cui

**Affiliations:** 1 School of Pharmacy, Changchun University of Chinese Medicine, Changchun 130117, China; wanglin@ccucm.edu.cn (L.W.); 24204701008@stu.ccucm.edu.cn (X.L.); 24204701004@stu.ccucm.edu.cn (H.L.); 2202030249@stu.ccucm.edu.cn (H.Z.); 2302020129@stu.ccucm.edu.cn (R.L.); 2202030341@stu.ccucm.edu.cn (S.X.); 2302020157@stu.ccucm.edu.cn (C.Z.); qiuzd@ccucm.edu.cn (Z.Q.); 2 College of Clinical Medicine, Changchun University of Chinese Medicine, Changchun 130117, China; lousl@ccucm.edu.cn; 3 Northeast Asian Institute of Traditional Chinese Medicine, Changchun University of Chinese Medicine, Changchun 130117, China

**Keywords:** osteosarcoma, miRNA, natural medicines, programmed cell death, m6A modification

## Abstract

miRNAs are small non-coding RNA molecules that play critical roles in the regulation of gene expression and have been closely associated with various diseases, including cancer. These molecules significantly influence the cell cycle of tumor cells and control programmed cell death (apoptosis). Currently, research on miRNAs has become a major focus in developing cancer therapies. Osteosarcoma, a malignant neoplasm predominantly occurring during adolescence and later in life, is characterized by a high propensity for metastasis. This review explores the role of miRNAs in the initiation and progression of cancer, highlighting their potential as predictive biomarkers for disease. It discusses the mechanisms by which natural products modulate miRNA activity to influence apoptosis, ferroptosis, and autophagy in osteosarcoma cells, aiming to identify new strategies for osteosarcoma treatment. Recent studies on how natural products regulate miRNAs to reduce tumor cell resistance to chemotherapy are also reviewed. Furthermore, the review elaborates on how natural products regulate m6A modifications to influence miRNA expression, thereby exerting antitumor effects. In this process, interactions between m6A modifications and miRNAs have been identified, with both jointly influencing tumorigenesis and cancer progression, offering a new perspective in osteosarcoma treatment. These approaches could help uncover novel regulatory mechanisms in osteosarcoma pathways and provide a theoretical foundation for developing new drugs and identifying novel therapeutic targets.

## Simple Summary

miRNAs are closely associated with the occurrence and development of osteosarcoma, playing a critical role in regulating the cell cycle and programmed cell death of osteosarcoma cells. This review elucidates the mechanisms by which miRNAs influence cancer progression and provides an in-depth analysis of the antitumor potential of natural compounds in regulating osteosarcoma cell apoptosis, ferroptosis, and autophagy through miRNA mediation. Additionally, natural products significantly reduce tumor cell chemoresistance and enhance the efficacy of chemotherapy by modulating miRNA expression. The findings also reveal that natural compounds can regulate miRNA expression via m6A modification, highlighting the important impact of the interplay between m6A modification and miRNAs on tumor development. These findings offer new potential directions and breakthroughs for osteosarcoma treatment.

## 1. Introduction

Osteosarcoma, a malignant primary bone tumor, is a rare malignant neoplasm originating from primitive mesenchymal cells within the skeletal system and commonly occurs during osteogenesis. The pathological characteristic of osteosarcoma is the formation of immature osteoid extracellular matrix [1]. Osteosarcoma arises from genetic mutations and the immune microenvironment, often leading to pain, swelling, and fractures. Approximately 3,600 new cases of osteosarcoma are diagnosed annually in Western countries, and ~1,000 in Asia [2]. Surgical tumor resection is one of the primary clinical treatment strategies, aiming to directly remove the affected tissue and halt the progression of the disease at its source [3]. Chemotherapy serves as an adjunctive treatment. Chemotherapeutic agents applied to eliminate potential residual cancer cells reduce the recurrence risk and enhance the overall therapeutic outcome, providing stronger support for patient recovery [4]. Nonspecific targeted therapy is an emerging strategy for osteosarcoma that disrupts key disease-associated cellular metabolic processes, modulates cell signaling pathways, and enhances immune system function. This approach increases the activity and efficacy of immune cells, enabling them to identify and destroy tumor cells [5]. While these methods have improved the survival rates of patients with osteosarcoma, several challenges persist. Surgical interventions may result in bone defects, whereas chemotherapy indiscriminately damages healthy cells. Additionally, nonspecific targeted therapy at high doses may cause significant adverse effects [6]. Osteosarcoma is also associated with substantial resistance to treatment and a tendency to metastasize and recur [7], leading to suboptimal outcomes in patients with advanced, metastatic, or recurrent disease. Therefore, further molecular, pathological, and physiological research is essential to reduce patient suffering and improve long-term survival.

The uncontrolled proliferation of tumor cells is a key factor in the progression of cancer [8]. Owing to genetic mutations or the dysregulation of growth mechanisms, tumor cells proliferate unchecked within the body, leading to tumor formation and the deterioration of bodily functions [9]. This process severely impacts patients’ health and can ultimately lead to death. Research has shown that different miRNAs play distinct roles in the initiation and progression of tumors. miRNAs that are overexpressed in cancer can function as oncogenes (such as miR-17-92 and miR-21), negatively regulating tumor suppressor genes [10] and controlling genes involved in cell differentiation and apoptosis, thereby promoting cancer development. In contrast, miRNAs that are under-expressed in cancer act as tumor suppressors (such as miR-15a and miR-16-1) and may inhibit cancer progression by regulating oncogenes or controlling genes involved in cell differentiation and apoptosis [11]. miRNAs can also influence the cell cycle and contribute to cancer prevention by regulating programmed cell death, effectively killing tumor cells. Additionally, miRNAs have shown promise in enhancing immune function and other therapeutic aspects.

Natural products are substances extracted from nature, including plants, herbs, and minerals, that often possess various pharmacological activities, such as anti-inflammatory, antioxidant, and antitumor properties [12]. Compared with chemically synthesized therapeutic products, natural products offer several advantages, including greater safety, fewer side effects, and a lower resistance risk. Consequently, natural products are frequently employed in conjunction with chemo-therapeutics to reduce the resistance developed by the body against chemo-therapeutics. In addition, natural products have a wide range of sources, are cost-effective, and possess diverse structural types. Therefore, natural products hold promising and extensive research potential in the development of novel anticancer products.

This review comprehensively delineates the molecular regulatory networks of miRNAs in osteosarcoma oncogenesis and systematically dissects the mechanisms through which natural products orchestrate miRNA-dependent signaling cascades in osteosarcoma therapeutics, with a particular emphasis on their capacity to circumvent chemotherapeutic resistance. Furthermore, this work elucidates the antitumoral efficacy of phytochemical interventions via m6A epitranscriptomic modifications, thereby establishing innovative therapeutic paradigms for osteosarcoma management.

## 2. The Role of miRNA Molecules in Tumor Biological Processes and Therapy

The identification of small non-coding RNA molecules (miRNAs) that regulate gene expression has been one of the most remarkable accomplishments in biology in recent years. It is widely believed that miRNAs, as posttranscriptional regulators of gene expression, induce mRNA degradation or translation inhibition by binding to the 3′ untranslated regions of specific messenger RNAs [13]. Moreover, there is a substantial body of literature indicating that miRNAs are pathologically dysregulated during cancer development. To date, miRNAs have been shown to be essential in modulating key protein-coding genes, signaling pathways, and biological processes associated with the pathogenesis of osteosarcoma.

**Figure 1:**
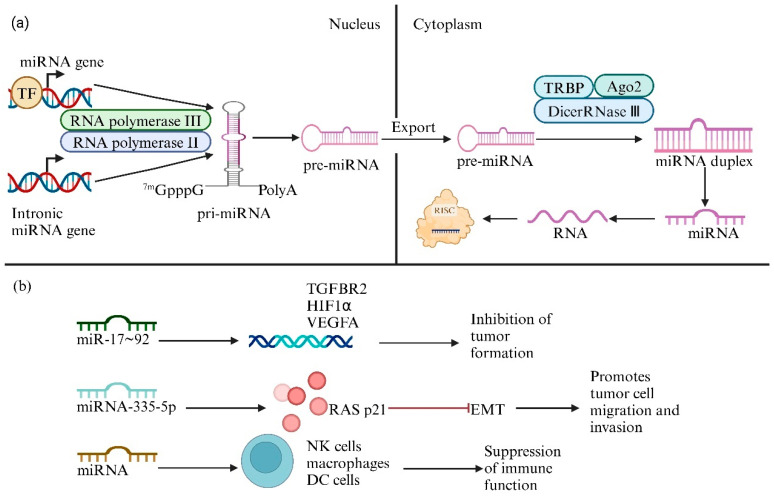
Biological processes of miRNAs. (**a**) The formation process of miRNAs. (**b**) The effects of miRNAs on tumor cells in vivo.

As shown in Figure 1a, most miRNA genes are transcribed by RNA polymerase II, whereas a small number are transcribed by RNA polymerase III. The primary processing of miRNAs occurs within the nucleus. Following the release of precursor proteins, miRNA hairpins are cleaved and processed by the RNase III enzyme Dicer, forming a complex with TRBP (TAR RNA-binding protein) and Ago2 (Argonaute 2) proteins. This complex converts the miRNA hairpin into a miRNA duplex. After the duplex unwinds, the mature miRNA is incorporated into the RNA-induced silencing complex (RISC), enabling it to execute gene silencing through its target mRNA [14]. The complementarity between nucleotides 6–8 at the 5′ end of a miRNA and the target mRNA influences the mechanism of mRNA inactivation. Complete complementarity leads to rapid cleavage and degradation of the mRNA, whereas partial complementarity blocks translation without causing immediate mRNA degradation, which relies on alternative silencing mechanisms.

Despite significant progress in understanding the biological origins and mechanisms of action of miRNAs, their potential applications in cancer therapy remain to be thoroughly explored. In 2002, the association between miRNAs and tumors was first revealed in chronic lymphocytic leukemia, with researchers observing the differential expression of miRNAs in various tumor tissues [15]. This discovery subsequently led to the understanding that miRNAs can function as oncogenes or tumor suppressors, playing crucial roles in tumor development and cancer therapy. Existing studies further indicate that miRNAs are deeply involved in various pathological processes of tumor cells. The growth and metastasis of tumors heavily rely on the nourishment provided by angiogenesis, and miRNAs can regulate multiple growth factors associated with angiogenesis, thereby influencing this process. For example, Ma et al. [16] demonstrated that the miR-17~92 cluster effectively inhibits tumor vascular formation by targeting pro-angiogenic genes such as TGFBR2, HIF1α, and VEGFA, resulting in insufficient nutrient supply that hinders the proliferation of tumor cells. Furthermore, miRNAs can regulate key proteins that influence cell migration and invasion, playing a preventative role in cancer metastasis. In the research conducted by Sun et al. [17], miRNA-335-5p has induced epithelial–mesenchymal transition (EMT) by downregulating the expression of RAS p21 protein activator 1 (RASA1), causing epithelial cells to lose their original polarity and tight junctions and gaining the migratory and invasive capabilities of mesenchymal cells, thereby promoting the invasion and metastasis of colorectal cancer (CRC) cells. Furthermore, aberrantly expressed miRNAs can promote tumor cell proliferation through various pathways, exacerbating the progression of cancer. As shown in Figure 1b, aberrantly expressed miRNAs can induce functional and phenotypic changes in natural killer (NK) cells, macrophages, and dendritic cells (DCs), even reversing the functions of these immune cells. This suppression of immune function allows tumor cells to evade surveillance by the innate immune system, further weakening the body’s ability to eliminate tumor cells [18].

Currently, numerous miRNA-based drugs have been evaluated in preclinical and clinical trials. For example, the miR-92a inhibitor MRG-110 underwent Phase 1 clinical trials in 2019 [19], demonstrating its potential to promote angiogenesis and improve wound healing in cardiovascular diseases, with confirmed safety and efficacy in humans. The miR-122 inhibitor miravirsen (SPC3649) forms oligomeric complexes with the HCV genome to increase HCV replication in hepatocytes. Phase 2 clinical trials evaluating miravirsen were completed in 2012 [20], although subsequent trial results remain unavailable. In the context of autosomal dominant polycystic kidney disease, Phase 1 trials evaluating the second-generation miR-17 inhibitor RGLS8429 were completed in 2022, showing considerable potential and ongoing subsequent clinical testing [21]. Furthermore, Phase 1 trials evaluating the therapeutic potential of the miR-29 mimic MRG-201 for treating keloids and fibrotic scars were completed in 2021, and the modified miR-29 mimic MRG-229 is currently undergoing preclinical testing for pulmonary fibrosis [22]. There is a substantial body of literature demonstrating the significant role of miRNAs in cancer therapy, suggesting that miRNAs may hold great diagnostic and prognostic value for patients with osteosarcoma; thus, miRNAs are expected to become potential therapeutic targets for treating osteosarcoma. 

In summary, miRNAs can target and regulate TGFBR2, HIF1α, and VEGFA to inhibit tumor angiogenesis. Moreover, miRNAs can modulate the expression of key proteins related to cell migration and invasion, thereby preventing cancer metastasis. However, abnormally expressed miRNAs can accelerate tumor cell deterioration through various pathways and even suppress immune function, weakening the body’s ability to eliminate tumor cells. Therefore, the expression levels of miRNAs may hold significant diagnostic and prognostic value for patients with osteosarcoma and have the potential to serve as promising therapeutic targets for osteosarcoma treatment.

## 3. Interaction Between Natural Products and miRNAs in Osteosarcoma Treatment

Natural products are widely sourced and possess diverse chemical components that can influence miRNA expression and function through various mechanisms, thereby regulating cellular biological behaviors [23]. Some natural products can directly interact with miRNA molecules. These products may bind to miRNAs via specific chemical domains, altering their conformational stability and subsequently affecting their binding affinity and specificity to target mRNAs [24]. These direct interactions effectively modulate the abundance of miRNAs, leading to changes in their intracellular concentrations and thereby regulating the expression of downstream genes.

In addition, natural products can indirectly influence the epigenetic regulation of miRNAs. Cell epigenetic regulatory mechanisms involve several key processes, including DNA methylation [25] and histone modifications [26]. Natural products can act on critical molecules involved in miRNA transcription or processing, such as DNA methyltransferases (DNMTs) and histone-modifying enzymes [27]. When natural products interact with these enzymes, their activity or ability to bind to DNA may be altered. For example, inhibiting DNMTs can reduce the methylation level of miRNA gene promoters, thereby promoting the expression of previously silenced genes [28]. 

Natural products regulate the expression and function of miRNAs within cells through both direct and indirect mechanisms, thereby influencing their biological behaviors. For example, in clinical treatment, triptolide downregulates the expression of oncogenic miRNAs, such as miR-17-92 and miR-106b-25, in a c-Myc-dependent manner. This effect leads to the upregulation of the miRNAs’ common target genes, including BIM, PTEN, and p21, subsequently inducing tumor cell death [29]. Salidroside can exert antitumor effects by regulating the miRNA–mRNA signaling axis, inducing apoptosis, autophagy, and cell differentiation and affecting the cell cycle in cancer cells. Huangqin glycoside can inhibit tumor cell proliferation, invasion, and metastasis by upregulating the expression of miR-126 and regulating the expression of related proteins [30]. In summary, the regulatory effects of natural products on miRNAs are highly important in fields such as oncology and neuroscience, opening new avenues and directions for therapeutic strategies.

Owing to their diverse therapeutic mechanisms, natural products have demonstrated unique advantages and achieved notable effects in treating osteosarcoma, as illustrated in Table 1. Studies have revealed that natural products interfere with cellular physiological processes through various pathways, leading to programmed cell death in osteosarcoma cells. Additionally, natural products inhibit osteosarcoma cell proliferation, differentiation, and metastasis. Furthermore, natural products possess antiangiogenic properties, disrupting the microenvironment for osteosarcoma angiogenesis. Additionally, natural products possess anti-angiogenic properties, capable of disrupting the angiogenic microenvironment of osteosarcoma, inhibiting the proliferation and migration of vascular endothelial cells, and reducing the blood supply to osteosarcoma, thereby limiting its growth and metastasis.

In summary, natural products can directly or indirectly influence the epigenetics of miRNAs and exert therapeutic effects on osteosarcoma by regulating various physiological processes.

## 4. Natural Products Mediate the Inhibition of Osteosarcoma Cell Proliferation, Differentiation, and Metastasis Through miRNA Regulation

Research on the biological processes of miRNAs has revealed that some miRNAs can influence the cell cycle of tumor cells, which can inhibit the proliferation, differentiation, and metastasis of tumor cells, effectively delaying tumor progression in patients and significantly extending their lifespan while increasing survival rates. Li et al. reported that miR-487a primarily promotes tumor cell metastasis by regulating the MAPK signaling pathway via the tumor suppressor gene SPRED2 and that it can also increase proliferation by affecting AKT signaling induced by PIK3R1. This finding opens new avenues for cancer treatment, specifically by silencing miR-487a to inhibit tumor cell proliferation and metastasis. It also provides a novel biomarker for monitoring cancer metastasis, specifically the upregulation of miR-487a expression [39,40]. Lin et al. [41] reported that the oncogene c-Myc is significantly upregulated in various types of cancer. Notably, the targets of c-Myc are not limited to miR-320a; it can also target other miRNAs, such as miR-24 [42], miR-145 [43], and miR-185-3p [44], with effects that are consistent with the in vivo functions of miR-310. Additionally, many studies have demonstrated that various natural products can influence biological processes, such as tumor proliferation, differentiation, and metastasis, by regulating miRNAs, as shown in Table 2. These findings offer new perspectives for the treatment of tumors such as osteosarcoma, suggesting that the modulation of miRNAs by natural products is a novel therapeutic approach.

Tara Jarboe et al. [45] reported that berberine can reduce the expression levels of hsa-miR-26b-5p, hsa-miR-125b-5p, hsa-miR-138-5p, hsa-miR-148a-5p, hsa-miR-152-5p, and hsa-miR-191-5p. Importantly, these miRNAs function as tumor suppressors; berberine effectively diminishes tumor cells’ migration and invasion potential through this mechanism. Triptolide, a natural product extracted from Tripterygium wilfordii, has been confirmed by Liu et al. [46] to significantly inhibit tumor cell proliferation, migration, and invasion by suppressing the expression of miR-146a. The research by Sun et al. [47] revealed another mechanism of action for curcumin—it induces cell cycle arrest by enhancing the expression levels of miR-34a, thereby diminishing the proliferative potential of tumor cells and ultimately inhibiting cancer progression. Örenlili Yaylagül et al. [48] revealed that baicalin indirectly inhibits the proliferation of osteosarcoma cells by modulating the expression of miR-25, which targets genes linked to the Wnt/β-catenin pathway. Additionally, Zhang et al. [49] confirmed that baicalein inhibits the proliferation and metastasis of osteosarcoma cells by promoting the expression of miR-183. Similarly, Yang et al. [50] studied resveratrol and revealed that it effectively prevents the invasion and migration of osteosarcoma cells by upregulating the expression of miR-328, which suppresses the activation of matrix metalloproteinase-2 (MMP-2). Davide Bertozzi [51] revealed a novel mechanism by which camptothecin inhibits cancer cell proliferation; acting as an inhibitor of DNA topoisomerase I, camptothecin can upregulate the expression of miR-17-5p and miR-155 in tumor cells, thereby suppressing the activity of HIF-1 alpha protein and effectively slowing the proliferation rate of cancer cells.

Overall, the aforementioned studies reveal the distinctive roles of various natural product monomers in regulating miRNA expression and highlight their potential applications in suppressing tumor cell proliferation, migration, and invasion. Therefore, utilizing natural products to mediate miRNAs in the suppression of tumor cell proliferation, differentiation, and metastasis offers a new direction for treating osteosarcoma and may advance research efforts in osteosarcoma therapy.

**Table 2:** The inhibitory effects of natural products mediated by miRNAs on tumors.

Natural Products	miRNAs	Cancer Type	Target	Effect	Cell Models	Reference
Pomegranate-derived peptide PG2	miR-339-5p	Leukemia	CDK2/miR-339-5p/caspase-3	Induce cell apoptosis	NB4 MOLT-4	[52]
Baicalein	miR-7	Gastric cancer	miR-7/FAK/AKT	Mediate cell proliferation, metastasis, and angiogenesis	HGC-27 SGC-7901 MGC-803 BGC-823	[53]
Elemene	miRNA-145-5p	Non-small cell lung cancer	miR-145-5p/MAP3K3/NF-κB	Suppress tumor growth	A549 H460 H322 H1299 293T	[54]
Schisandrin B	miR-708-5p	Osteosarcoma	PI3K/AKT	Inhibit cell viability and migration, induce cell apoptosis	SaOS2 U2OS	[55]
Rutin	miR-877-3p	Pancreatic cancer	Bcl-2	Induce cell apoptosis	PANC-1 SW1990 MIA PaCa-2	[56]
Homoharringtonine	miR-18a-3p	Breast cancer	miR-18a-3p/AKT/mTOR	Suppress cell growth and promote apoptosis	MDA-MB-231 MCF-7 T47D HCC1937 MCF-10A	[57]
Andrographolide	miR-21-5p	Breast cancer	NF-κB/miR-21-5p/PDCD4	Suppress the growth and metastasis	MCF-7	[58]
Maackiain	miR-374a	Triple-negative breast cancer	miR-374a/GADD45A	Promote cell proliferation, migration, and invasion	MDA-MB-231 BT549 FBS	[59]
Ursolic acid	miR-140-5p	Colorectal cancer	TGF-β3	Inhibit proliferation and cell cycle and promote apoptosis	SCSP-5032	[60]

## 5. Natural Products Mediate miRNA to Promote Apoptosis in Osteosarcoma Cells

**Figure 2:**
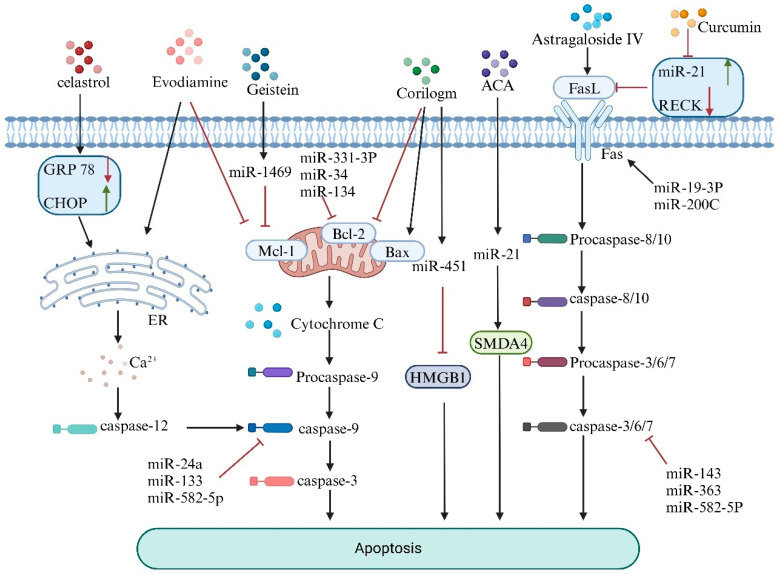
Mechanisms by which natural products regulate miRNAs to promote apoptosis.

Apoptosis is a classic form of programmed cell death [61]. Under normal physiological conditions, the body can eliminate aging or abnormal cells through apoptosis, thereby maintaining homeostasis. In contrast, the dysregulation of apoptosis in pathological states disrupts the balance between cell proliferation and death, leading to detrimental effects on the organism and resulting in a series of diseases, including the proliferation and metastasis of osteosarcoma [62]. Recent studies have shown that natural products can induce apoptosis in osteosarcoma cells by regulating miRNAs to influence apoptosis-related proteins and pathways, such as the mitochondrial apoptosis, death receptor, and endoplasmic reticulum pathways [63], as illustrated in Figure 2.

The mitochondrial apoptosis pathway is governed mainly by the ratio of anti-apoptotic genes (Bcl-2) to pro-apoptotic genes (Bax) within the Bcl-2 gene family [64]. When cells are subjected to external stimuli, the ratio of Bcl-2/Bax decreases, leading to increased mitochondrial membrane permeability and promoting the release of apoptosis-related proteins, such as cytochrome C, from the mitochondria. This process activates the caspase family of proteins, triggering apoptosis in osteosarcoma cells [65]. Inhibitors of apoptosis proteins (IAPs), such as XIAP and BIRC2, can also regulate apoptosis by inhibiting caspases [66]. Xiao et al. [67] revealed that the expression of miR-139-5p is greater in normal human osteoblasts than in the osteosarcoma cell line U2OS. The overexpression of miR-139-5p inhibited apoptosis in U2OS cells, whereas the suppression of miR-139-5p had the opposite effect. Resveratrol, a natural polyphenolic compound, can trigger apoptosis in osteosarcoma cells by downregulating the miR-139-5p/Notch-1 signaling pathway.

Jia et al. [68] determined that the active component of the natural product corilagin upregulates the expression of proapoptotic proteins (such as Bak), downregulates Bcl-2 expression, and activates caspase-3, thereby triggering the mitochondrial apoptosis pathway and promoting tumor cell apoptosis. Additionally, they reported that corilagin could significantly reduce the mRNA and protein levels of HMGB1 by targeting and enhancing the expression of miR-451, ultimately leading to tumor cell apoptosis.

The mechanism of the death receptor pathway involves the binding of death ligands (such as FasL) to death receptors on the cell surface (such as Fas, a member of the tumor necrosis factor (TNF) receptor family), which transmits death signals into tumor cells, ultimately leading to their apoptosis [63]. However, Fas is expressed at low levels on the surfaces of many tumors, preventing its binding with FasL and allowing the tumor to evade damage from T lymphocytes [69]. Various natural products have been found to restore the expression of Fas on the surface of tumor cells, thereby exerting therapeutic effects against tumors [70]. Hu et al. [71] reported that astragaloside IV combined with cisplatin markedly increases the expression of Fas and FasL, promoting osteosarcoma cell apoptosis through a caspase-dependent Fas/FasL signaling pathway.

Studies have indicated that miR-21 is upregulated in osteosarcoma, with the retinoblastoma-inducible cysteine-rich Kazal-type protein (RECK) identified as a target of miR-21 [72]. According to Zhou et al. [73], the classical antitumor natural compound curcumin can regulate the expression of miR-21/RECK in osteosarcoma, thereby promoting apoptosis in these cells and successfully establishing a novel therapeutic approach for the treatment of this cancer. Additionally, Zhang et al. [74] found that miR-21 inhibits the binding of Fas and FasL, leading to the hypothesis that curcumin may also induce apoptosis in osteosarcoma cells by downregulating miR-21 and activating the death receptor pathway.

Furthermore, under pathological conditions, cells enter a state of endoplasmic reticulum stress (ERS) [75], which triggers the unfolded protein response (UPR). This response aims to clear misfolded proteins and restore cellular function [76]. However, when ER function is severely impaired, UPR can activate apoptotic signaling cascades by triggering caspase activation, specifically caspase-12, leading to apoptosis [77]. ER stress-related proteins such as GRP78 and the apoptosis-associated protein CHOP are key transcription factors specific to the ER stress response [78]. Wang et al. [79] found that combining triptolide with cisplatin reduced GRP78 protein expression while increasing CHOP protein levels, effectively inducing osteosarcoma cell apoptosis through the ER stress pathway.

Evodiamine is an alkaloid extracted and isolated from the fruit of Evodia rutaecarpa [80]. In osteosarcoma, evodiamine increases the expression levels of Bax, caspase-3, and poly ADP–ribose polymerase (PARP), a DNA repair enzyme and substrate for caspases, which are core members of the apoptosis pathway [81]. Moreover, PARP downregulates the expression of Bcl-2 and survivin. These effects are mediated through the mitochondrial apoptosis pathway, thereby exerting anti-osteosarcoma activity. Additionally, evodiamine induces cell cycle arrest at the G2/M or G0/G1 phase and modulates the PTEN/PI3K/Akt signaling pathway, rendering it inactive, as does the inhibition of the Wnt/β-catenin signaling cascade. These mechanisms collectively contribute to its therapeutic potential in osteosarcoma treatment [82].

In summary, various natural products can regulate miRNA expression, either by upregulation or downregulation, to modulate the ERS status and the Bcl-2/Bax signaling, death receptor, and Fas/FasL mitochondrial signaling pathways. These mechanisms induce osteosarcoma cell apoptosis, thereby achieving therapeutic effects.

## 6. Natural Products Mediate miRNAs to Promote Ferroptosis in Osteosarcoma Cells

Ferroptosis is a novel form of programmed cell death dependent on iron [83]. The primary mechanism underlying ferroptosis involves the susceptibility of polyunsaturated fatty acid-containing phospholipids in the cell membrane to lipid peroxidation under conditions of iron abundance and elevated reactive oxygen species. The accumulation of lipid peroxides within the membrane eventually causes rupture, thereby inducing cell death [84]. This process is often associated with the reduced activity of glutathione peroxidase 4 (GPX4), a key antioxidant enzyme [85]. Emerging research has revealed that various miRNAs function as either oncogenes or tumor suppressors [86], influencing ferroptosis pathways by regulating lipid metabolism [87], iron homeostasis [88], the glutathione system [89], and oxidative stress responses [90]. By modulating miRNA activity to trigger ferroptosis, natural products offer promising therapeutic strategies for osteosarcoma treatment.

**Figure 3:**
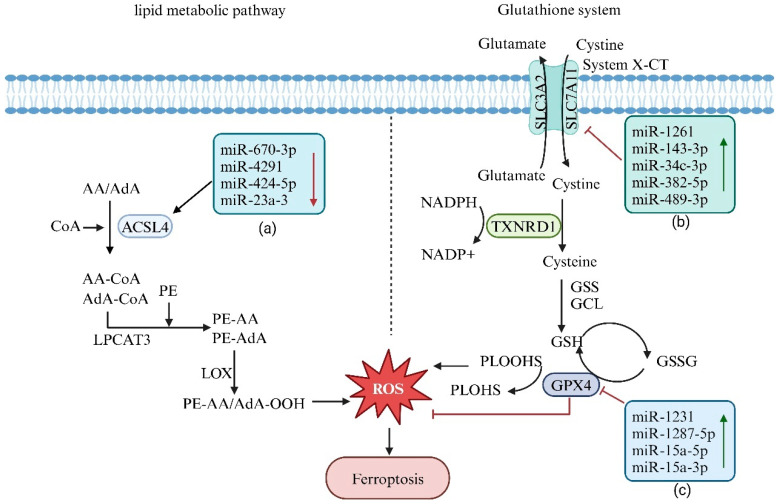
The regulatory role of miRNA in ferroptosis. (**a**) Downregulating the expression of miRNAs that inhibit ferroptosis leads to the accumulation of lipid peroxides, thereby inducing ferroptotic cell death. (**b**) Upregulating the expression of ferroptosis-inducing miRNA inhibits x-CT expression, resulting in increased ROS levels and triggering ferroptotic cell death. (**c**) Upregulating the expression of ferroptosis-inducing miRNA suppresses GPX4 activity, leading to elevated ROS levels and initiating ferroptotic cell death.

The regulation of ferroptosis-related lipid metabolic pathways by miRNAs is primarily achieved through their influence on genes associated with lipid metabolism [91], as illustrated in Figure 3. For example, miRNAs can target and modulate the expression of ACSL4 (long-chain acyl-CoA synthetase 4) and LPCAT3 (lysophosphatidylcholine acyltransferase 3). ACSL4 catalyzes arachidonic acid (AA) and adrenic acid (AdA) into arachidonoyl-CoA (AA-CoA) and adrenoyl-CoA (AdA-CoA). Subsequently, LPCAT3 mediates the esterification of these intermediates with phosphatidylethanolamine (PE) on the cell membrane, generating AA-PE and AdA-PE. These products are then oxidized by lipoxygenases (LOX) to produce lipid peroxides [92]. By increasing the expression levels of ACSL4 and LPCAT3, miRNAs facilitate the accumulation of lipid peroxides, ultimately leading to ferroptosis [93].

Zhang et al. [94] elucidated that arachidonate 15-lipoxygenase (ALOX15) augments the production of lipid peroxides in gastric cancer, whereas exosomal miR-522 serves as a potential antagonist of ALOX15. A decrease in miR-522 levels results in increased ALOX15 and reactive oxygen species (ROS) levels, ultimately precipitating ferroptosis in osteosarcoma cells.

In the regulation of the glutathione cycle, miRNAs mediate ferroptosis by modulating ferroptosis-inhibiting genes, such as solute carrier family 7 member 11 (SLC7A11), solute carrier family 3 member 2 (SLC3A2), and glutathione peroxidase 4 (GPX4). miRNAs can suppress or promote ferroptosis by upregulating or downregulating the expression of these genes [95]. Studies have shown that miR-1261, miR-143-3p, miR-34c-3p, miR-382-5p, and miR-489-3p induce ferroptosis in cancer cells by targeting and downregulating the expression of the ferroptosis inhibitor SLC7A11 [96]. Similarly, miR-302a-3p and miR-4735-3p trigger ferroptosis in lung and renal cancer cells by downregulating SLC40A1 expression [96]. In general, miRNAs that suppress GPX4 expression exhibit significant potential to induce ferroptosis. This finding has been validated in studies on miR-324-3p in prostate cancer PC-3 cells [97] and miR-1291 in colon cancer cells [98]. Furthermore, hyperglycemia has been shown to downregulate miR-223-3p expression, thereby reducing xCT and GPX4 expression and inducing ferroptosis in glomerular endothelial cells. This finding identifies miR-223-3p as a ferroptosis-suppressing miRNA [99].

Zhang et al. demonstrated that oridonin can downregulate the expression of cl-caspase3, SLC7A11, BAX, GPX4, and FTH1 proteins and their corresponding mRNAs while upregulating the expression of Bcl-2 and ACSL4 in 143B and U2OS cells. In addition, oridonin facilitates the accumulation of reactive oxygen species (ROS) and Fe^2+^, thereby confirming its role in inducing apoptosis and ferroptosis in osteosarcoma cells. Consequently, oridonin has emerged as a promising and safe therapeutic agent for the treatment of osteosarcoma [100].

Chen et al. [101] demonstrated that artemisinin (ART) products, including dihydroartemisinin (DAT), can enhance the sensitivity of osteosarcoma cells to ferroptosis by depleting cysteine and suppressing GPX4 activity. Another study revealed that a high-fat diet enriched with quercetin promotes the expression of miR-125b-5p in mice [102]. Furthermore, apigenin, a natural flavonoid with potent antineoplastic activity, has shown efficacy in inhibiting the proliferation of various tumor cells, including human osteosarcoma cells [103]. Evidence indicates that apigenin induces ferroptosis in tumor cells by reducing intracellular GSH levels, subsequently decreasing the protein expression of SLC7A11 and GPX4. This process effectively inhibits tumor proliferation and restrains growth [104]. Thus, apigenin emerges as a promising and efficacious therapeutic candidate for the treatment of osteosarcoma.

In summary, this paper highlights the correlation between natural products, miRNAs, and autophagy. Natural products exert dual regulatory effects on osteosarcoma cell autophagy by modulating miRNA expression, thereby demonstrating anti-osteosarcoma activity. This study offers new insights into the development of novel, efficient, and low-toxicity therapeutic strategies for osteosarcoma.

## 7. Natural Products That Modulate miRNAs Influence Autophagy in Osteosarcoma Cells

Autophagy is a unique cellular self-protection mechanism [105]. In osteosarcoma, autophagy acts as a “double-edged sword” [106]. On the one hand, during the early stages of tumor formation, under stress conditions such as hypoxia and metabolic disturbances, osteosarcoma cells transport damaged organelles, proteins, microbes, and viruses into intracellular double-membrane vesicles, followed by lysosomal transfer and subsequent degradation. This process inhibits inflammatory responses, creating an environment unfavorable for osteosarcoma cell growth [107]. On the other hand, in the later stages of the tumor, autophagy can often increase the survival rate of tumor cells under stress and maintain tumor stem cells, thereby promoting tumor growth [108]. Therefore, regulating autophagy can be a strategy for treating osteosarcoma. The dysregulation of autophagy in human tumor cells is also influenced by miRNAs [109]. Thus, the regulation of autophagy by miRNAs holds potential for cancer therapy. As shown in Table 3, numerous studies have demonstrated that miRNAs play dual roles in the regulation of autophagy in tumor cells [110]. Natural products can modulate miRNAs to either inhibit or promote autophagy as a therapeutic approach for osteosarcoma.

Existing evidence suggests that certain miRNAs, such as miR-488-3p, can inhibit the proliferation of osteosarcoma cells by enhancing autophagy [111]. Oleuropein (OLEU) is a naturally active compound derived from olive trees with anti-proliferative properties against osteosarcoma that can increase the cytotoxicity of adriamycin (ADR) by interfering with ADR-induced autophagy. OLEU is an edible product with no toxic side effects, making it a potential adjuvant therapy for osteosarcoma [112]. According to previous reports, the anticancer mechanism of triptolide involves inducing ER stress by increasing the accumulation of ROS within tumor cells, thereby promoting protective autophagy in tumor cells treated with triptolide [113]. Additionally, it has been confirmed that the upregulation of miR-141 expression can reduce ATG5 and LC3-II expression, significantly inhibiting autophagy in osteosarcoma cells [114].

In summary, there are substantial correlations among natural products, miRNAs, and autophagy. Natural products exert dual regulatory effects on osteosarcoma cell autophagy by modulating miRNA expression, demonstrating anti-osteosarcoma activity. The aforementioned studies offer new insights into the development of novel, efficient, and low-toxicity therapeutic strategies for osteosarcoma.

**Table 3:** miRNAs that modulate autophagy for osteosarcoma treatment.

miRNA	Expression	Expression Effect	Target Gene	Type of Cell Line	Reference
miR-375	up	Suppress Cell Proliferation and Autophagy	ATG2B	hFOB1.19 U2OS MG63	[115]
miR-19	up	Promote Cell Proliferation, Invasion, Migration, and EMT	SPRED2	hFOB MG-63 SOSP-9607 Saos-2 U2OS	[116]
miR 22	up	Inhibit Autophagy and Induce Apoptosis	ATG5 beclin1 LC3	MG-63, U2OS, Saos2	[117]
miR-579-3p	down	Promote Autophagy	MSH6	U-2OS MG63	[118]
miR-495-3p	up	Induce Mitophagy and Apoptosis	Sphk1	BEAS2B A549 H1299	[119]
miR-29a-3p	up	Aggravate Apoptosis; Dampen Cell Proliferation, Colony Formation, Migration, and Invasion; and Promote Autophagy	IGF1	143B MG-63 HOS SJSA-1 hFOB 1.19	[120]
miR-488-3p	down	Repress Malignant Behaviors and Facilitate Autophagy	Neurensin-2	U2OS Saos2 OS 99-1	[111]
miR-22	up	Suppress Proliferation and Promote Sensitivity; Mediate Autophagy	MTDH	MG-63	[121]
miR-193b	up	Induce Autophagy and Apoptosis	FEN1	MG-63 U2OS 143B	[122]
miR-506-3p	up	Suppress Cell Invasion	SPHK1	MG63 143B MNNG/HOS SaOS-2 U-2OS	[123]

## 8. Natural Products Modulate miRNA to Regulate Drug Resistance in Tumor Cell

A major challenge in cancer treatment is the side effects of chemotherapeutic products and the resistance developed by cancer cells after prolonged exposure [124]. Studies indicate that the majority of cancer patient deaths are due to drug resistance [125]. In various malignancies, miRNAs can be encapsulated in exosomes and transferred between cells, influencing protein expression [126] and regulating drugs in the tumor. In recent years, the active components of natural products have shown promise in reducing chemotherapy side effects, improving drug resistance, and enhancing tumor sensitivity to products due to their unique chemical structures and biological activities [23]. Researchers are exploring the combination of natural products with chemotherapy to inhibit cancer cell resistance, thereby effectively improving patient outcomes and survival rates.

Chemotherapy is a key component of comprehensive treatment for osteosarcoma. The mechanisms of chemotherapy resistance are complex and involve multiple factors, including disturbances in drug accumulation mechanisms within cells, intracellular detoxification, apoptosis, DNA damage repair, signal transduction, and the tumor microenvironment [127]. Therefore, a deep understanding of resistance mechanisms and their associated miRNAs is crucial for developing effective therapeutic strategies.

miRNAs play dual roles in regulating the sensitivity of tumor cells to chemotherapeutic drugs. On the one hand, they can contribute to drug resistance by modulating drug transporters to reduce intracellular drug concentrations, affecting apoptosis pathways to enable tumor cells to evade apoptosis and participate in DNA damage repair mechanisms [128]. On the other hand, miRNAs can inhibit drug resistance-associated signaling pathways and regulate the tumor microenvironment, thereby increasing the sensitivity of tumor cells to chemotherapy [129]. The differential expression of miRNAs is closely associated with drug resistance in various tumors. Studies have shown that the increased expression of miRNA-216b can regulate the JMJD2C/HIF1α/HES1 signaling axis in osteosarcoma cells, thereby increasing cisplatin-induced apoptosis and effectively treating osteosarcoma [130]. Doxorubicin (DOX) is a commonly used anthracycline antitumor drug for osteosarcoma treatment, but prolonged use can lead to resistance and adverse effects such as renal impairment, myelosuppression, and neurotoxicity. Therefore, alternative therapeutic strategies are urgently needed. For example, in osteosarcoma tissues and DOX-resistant osteosarcoma cells, miR-198 targets and upregulates ABCB1 expression, increasing the sensitivity of osteosarcoma cells to doxorubicin and thereby enhancing drug efficacy [131]. Another study revealed that the upregulation of miR-26a-5p can further target FTH1 mRNA and reduce FTH1 translation efficiency, significantly increasing the sensitivity of osteosarcoma cells to doxorubicin and cisplatin, thus improving drug efficacy [132].

Currently, many natural products can inhibit tumor drug resistance by modulating miRNAs, as shown in Table 4. Natural products exhibit multifaceted roles in overcoming chemotherapy resistance. Some natural drugs activate apoptosis signaling pathways in tumor cells, increasing the sensitivity of leukemia and breast cancer cells to chemotherapy by regulating mitochondrial function, activating caspase family proteases, and other mechanisms [133]. Other natural products reduce resistance by interfering with cancer cell cycle progression and inhibiting excessive proliferation. By modulating the expression of cyclins and kinases, these products induce cancer cell arrest at specific cell cycle stages, thereby enhancing the efficacy of chemotherapy in colorectal cancer, pancreatic cancer, and other cancers [134]. Furthermore, quercetin can inhibit the activity of transporters, reversing resistance and increasing chemical drug concentrations in drug-resistant breast cancer and gastric cancer cell lines [135]. Additionally, natural products can intervene in aberrantly activated signaling pathways associated with chemotherapy resistance in tumor cells. For example, studies have demonstrated that luteolin reduces osteosarcoma chemotherapy resistance by upregulating miR-384 expression and inhibiting the PTN/β-catenin/MDR1 signaling axis, with exosomal miR-384 being transferred to recipient osteosarcoma cells, thereby inhibiting doxorubicin resistance [136]. Zhang et al. [137] reported that quercetin enhances the sensitivity of osteosarcoma cells to cisplatin by modulating the miR-217-KRAS axis. MiR-217 targets KRAS in 143B cells, inhibiting their proliferation, migration, and invasion. Antagonists knocking down miR-217 had the opposite effect, indicating that the quercetin regulation of miR-217 can reverse doxorubicin resistance. Bufalin can induce the overexpression of miR-148a to target and regulate DNMT1, inhibiting the stemness of cancer stem cells in primary osteosarcoma cells to reduce resistance [138]. Natural products regulate miRNA expression and influence tumor cell survival, proliferation, apoptosis, and the expression of drug resistance-related proteins. Various experimental studies on cancer have shown synergistic effects with chemotherapy drugs, offering new hope and directions for overcoming chemotherapy resistance in tumors.

However, the active components of many natural products are complex and diverse, and their full identification remains incomplete. Moreover, their mechanisms of action are not yet fully understood, presenting significant risks and uncertainties for clinical application. While some components are known, their large quantities make manual screening challenging. Fortunately, the rapid advancement of AI technologies has provided strong support for natural product screening. For example, Aldas-Bulos et al. [139] developed AI methods for the rational application of traditional drug bioactivity by capturing special structural molecules and designing combinations or targeting selectivities. Li et al. [140] screened 150 molecules for experimental validation, identifying new CTSL inhibitors via AI and experimental methods. Qi et al. [141] screened nine natural products and demonstrated that crocin could directly bind to and inhibit MMP2 activity through biolayer interferometry and AI/bioinformatics techniques. Although AI has played a significant role in screening natural products and resolving various challenges, it is currently only predictive and cannot fully replace the role of humans. Instead, AI is an auxiliary tool for preliminary screening and analysis, providing directions and references for subsequent research. It is believed that, with more clinical trials to refine and validate the medicinal mechanisms of natural products, the full potential of AI will be harnessed to clarify the components of natural products and optimize combinations with certain therapies to improve osteosarcoma treatment, advancing both the research and application of natural medicines.

**Table 4:** Natural products that modulate miRNA to inhibit drug resistance in tumor cells.

Natural Products	Tumor Type	Corresponding Products	miRNAs	Dysregulation	Pathway/Target	Reference
DET	CC	5-FU	miR-205/Bcl2	Upregulated	miR-205/Bcl2	[142]
Curcumol	TNBC	DOX	miR-181b-2-3p	Upregulated	miR-181b-2-3p-ABCC3	[143]
Quercetin	CRC	5-FU	miR-27a	Downregulated	miR-27a/Wnt/β-catenin	[144]
Terpenoids	CC	5-FU	miR-495-3p	Upregulated	P-gp	[145]
Rutin	HCC	SFN	miRNA-590-5P	Upregulated	BANCR/miRNA-590-5P/OLR1	[146]
Cur	CRC	DDP	miR-137	Upregulated	GLS	[147]
AC	CC	5-FU	miR-142-3p	Upregulated	ABCG2	[148]
Tan IIA	CRC	OXA	miR-30b-5p	Upregulated	microRNA-30b-5p/AVEN	[149]
Cur	CRC	OXA	miR-409-3p	Upregulated	ERCC1	[150]
Y6	HCC	OXA	MiR-338-3p	Upregulated	MiR-338-3p/HIF-1α/TWIST	[151]
Cur	AML	DOX	miR-20a-5p	Downregulated	lncRNA HOTAIR/miR-20a-5p/WT1	[152]
G-Rg3	GC	DDP	miR-429	Upregulated	SOX2 PI3K/AKT/mTOR	[153]

Abbreviations: miRNA, microRNA; DET, Deoxyelephantopin; CC, Colon Cancer; 5-FU, 5-fluorouracil; TNBC, Triple-Negative Breast Cancer; DOX, Doxorubicin; CRC, Colorectal Cancer; HCC, Hepatocellular Carcinoma; SFN, Sorafenib; DDP, Cisplatin; AC, Antrodia Cinnamomea; OXA, Oxaliplatin; Tan IIA, Tanshinone IIA; Cur, Curcumin; HCC, Hepatocellular Carcinoma; AML, Acute Myeloid Leukemia; G-Rg3, Ginsenoside-Rg3; GC, Gastric Cancer.

## 9. The Potential of Natural Products to Exert Antitumor Effects by Targeting m6A Modifications

Recent studies have revealed that natural products may modulate tumorigenesis and cancer therapy by influencing RNA modification processes [154]. RNA modification refers to chemical alterations that affect the function, stability, processing, and interactions of RNA molecules [155]. More than 170 types of RNA modifications have been identified thus far, among which N6-methyladenosine (m6A) is the most abundant and significant [156]. m6A is a dynamic and reversible modification catalyzed by methyltransferases, demethylases, and binding proteins. It plays a crucial regulatory role and is involved in various cellular processes and pathological mechanisms of diseases [157]. Within cells, m6A modification relies on auxiliary factors such as writer proteins (METTL3, METTL14, and WTAP), eraser proteins (FTO and ALKBH5), and reader proteins (YTH domain family proteins and elF3) that regulate RNA processing, decay, and translation. These proteins are aberrantly expressed in various cancers and exert oncogenic or antitumor effects by influencing the expression of specific genes [158]. Increasing evidence suggests that m6A modification can regulate the expression and function of miRNAs, thereby affecting cellular immunity, gene expression, proliferation, differentiation, and apoptosis [159]. Proteins associated with m6A modifications facilitate pri-miRNA processing into pre-miRNAs and subsequent steps in miRNA maturation [160]. For instance, METTL3 collaborates with HNRNPA2B1 to recruit DGCR8 through m6A modification, enhancing pri-miRNA recognition and processing [161]. A study by Ma et al. [162] demonstrated that miR-126, a tumor suppressor involved in metastasis, is a downstream target of the m6A writer METTL14. METTL14 positively regulates pri-miR-126 maturation via a DGCR8-dependent mechanism, thereby upregulating miR-126 expression and inhibiting HCC migration and invasion. This finding underscores m6A modification as a mediator of miRNA regulation and highlights the interaction between METTL14 and miRNAs as a promising target for cancer therapy [163].

Moreover, studies have shown that miRNAs can bind to the 3′-UTRs of mRNAs encoding m6A-related proteins (e.g., METTL3, FTO, and YTHDF1), inhibiting their translation and regulating m6A modification in vivo [164]. For example, miR-145 exhibits antitumor activity across various cancers, such as colorectal cancer, where it targets the 3′-UTR of ERG mRNA [165]. Yang et al. [166] found that YTHDF2, an m6A reader protein, typically induces mRNA degradation upon recognizing m6A sites, thereby lowering m6A levels. In HCC, miR-145 targets the 3′-UTR of YTHDF2 mRNA, reducing its expression, increasing mRNA m6A levels, and inhibiting HCC proliferation while promoting apoptosis. Li et al. [167] extended these findings, revealing a double-negative feedback loop between miR-145 and YTHDF2, where YTHDF2 suppresses miR-145 expression, thereby hindering tumor growth.

These findings illustrate a reciprocal regulatory relationship between miRNAs and m6A modifications. Natural products can modulate m6A-related proteins, indirectly influencing miRNA expression, or mediate the miRNA regulation of m6A modifications, thereby affecting physiological processes and disease progression. Future research should harness advanced technologies, such as gene editing, RNA interference, and artificial intelligence [168], to delve deeper into how natural products regulate miRNA-mediated m6A modifications for cancer therapy. Screening for safe, low-toxicity, and efficient natural products as bidirectional regulators of RNA modification and miRNAs offers a novel avenue for clinical cancer treatment.

## 10. Discussion

Over the past few decades, treatments for osteosarcoma have been continuously updated as scientists strive to discover more effective methods. Recently, natural products have gained international recognition for their multiactivity, multitarget, and multipathway characteristics. Some traditional Chinese medicine components have shown significant pharmacological effects on tumor cells and play irreplaceable roles in cancer treatment [169]. This review focused on how natural products can influence osteosarcoma-related biological processes by targeting and regulating miRNAs, which are crucial in osteosarcoma cell proliferation, metastasis, and chemotherapy resistance. These products can reduce the physiological pain and side effects caused by conventional therapies. This treatment approach can be applied in early-stage osteosarcoma to trigger intrinsic mechanisms that eliminate cancer cells and prevent their proliferation. However, owing to the complex chemical composition and numerous targets of natural products, their complete mechanisms of action are not yet fully understood. These products can affect multiple pathways and functions within the body, with some providing therapeutic effects. In contrast, others may cause adverse reactions (such as insomnia, palpitations, gastrointestinal mucosal damage, and kidney damage [170]). Several strategies can be employed to limit the pleiotropic effects of natural products and increase their medicinal value. First, targeted formulations are effective. By encapsulating natural products in nanoparticles or binding them with specific targeting ligands, delivery to specific cells or tissues can be improved, reducing the effects on other normal cells and thus minimizing pleiotropy [171]. Second, biomarker identification is crucial. By identifying biomarkers that respond to certain natural products, personalized treatment can be achieved, reducing the risk of side effects [172]. Finally, high-throughput screening can be used to discover drugs that can be combined with natural products to identify combinations that enhance efficacy and reduce side effects [173]. These strategies aim to mitigate the adverse effects of the pleiotropy of natural products, improve their safety and efficacy in treatment, and provide multiple avenues for better utilization of natural products.

Additionally, natural products can be combined with traditional anticancer drugs to enhance the sensitivity of osteosarcoma cells to these drugs, reducing chemotherapy resistance while increasing efficacy [174]. However, natural products often have a low therapeutic index, with a narrow margin between effective and toxic doses [175]. This means severe side effects may occur even at doses below the effective level. Variations in individual genetic traits, health conditions, and concurrent medications can further influence responsiveness, increasing the risk of side effects and hindering the development of natural products. There are numerous potential risks when natural products are used in combination with radiotherapy or chemotherapy. For example, interactions during metabolism may alter the rate and extent of drug metabolism [176], or certain components may form complexes with radiotherapy or chemotherapy drugs in the gastrointestinal tract, interfering with absorption and altering drug distribution within the body. This effect can lead to decreased drug concentrations in tumor tissues and increased concentrations in other tissues, exacerbating toxic side effects [177]. However, by implementing the precise optimization of individualized dosing [178]; regularly monitoring and evaluating patient biomarkers [179], blood-related indicators, and liver and kidney functions; carefully selecting compatible drug combinations; and applying adjunct therapies appropriately, the harmful effects of combining natural products with radiotherapy or chemotherapy can be effectively mitigated. This approach enables the development of safer and more effective treatment plans tailored to individual patients, advancing the combined treatment model in a more favorable direction.

RNA translational modifications play crucial roles in cancer progression. Rapid tumor cell proliferation requires abundant ribosome production [180] and increased translation rates, necessitating coordinated gene expression across transcription and translation stages to drive protein synthesis. Among numerous RNA modifications, m6A has emerged as a focal point in cancer research [181]. This review highlighted studies on m6A-related proteins, identifying them as potential targets for natural products in anti-osteosarcoma therapy and providing new research directions.

While natural products show potential in targeting miRNAs for cancer therapy, they also present several limitations. The specific active components of natural products and their precise interaction mechanisms with miRNAs are often difficult to define. For instance, many plant extracts contain many secondary metabolites that may have synergistic or antagonistic effects [182]; thus, it is challenging to identify which component truly has a critical impact on miRNA. This complexity affects the stability and predictability of therapeutic outcomes. Additionally, some natural products may have poor bioavailability due to their molecular structure or polarity [183], leading to poor absorption in the gastrointestinal tract or rapid degradation and excretion during metabolism [184]. This results in an insufficient compound concentration entering the bloodstream to effectively target miRNAs in cancer cells, limiting their therapeutic potential. The lack of standardized production and quality control of natural products means that different batches may vary significantly in efficacy when used to target miRNA for cancer treatment, increasing clinical risks and research challenges. Furthermore, cancer is a complex disease involving multiple gene and signaling pathway abnormalities [185]. Relying solely on natural products to target miRNAs may not comprehensively and effectively inhibit tumor cell growth and metastasis [186]. Tumor cells often evade treatment through various mechanisms, such as activating compensatory signaling pathways or altering miRNA expression profiles. Therefore, the use of natural products alone to modulate miRNAs may be insufficient to address the complexity and heterogeneity of cancer. Combining these products with other therapeutic approaches is necessary; however, optimal combination therapy strategies remain to be further explored.

## 11. Conclusions and Outlook

This review summarized recent research findings on the anti-osteosarcoma mechanisms of various active components in natural products on the basis of miRNA regulation. These findings highlight the role of natural products in osteosarcoma prevention and treatment through the targeted regulation of miRNAs and underscore their significant effectiveness in reducing tumor cell drug resistance. Furthermore, these findings provide a theoretical basis for optimizing combinations of natural products and chemotherapeutic agents. Additionally, the interaction between m6A modifications and miRNAs offers a novel direction for osteosarcoma treatment, with the potential to overcome current therapeutic limitations. As natural products can regulate miRNA expression, they exhibit powerful multitarget regulatory capabilities, enabling precise intervention in abnormal physiological and metabolic processes of tumor cells, tumor microenvironment reshaping, and cell proliferation, differentiation, and metastasis inhibition, potentially comprehensively suppressing osteosarcoma progression. In conclusion, natural products hold promise as more effective therapeutic strategies for osteosarcoma, providing valuable theoretical support for its treatment and future research.

In future medical research, as the critical role of miRNAs in the progression and pathogenesis of human diseases becomes increasingly clear, the exploration of natural products using miRNAs to treat osteosarcoma holds significant potential. Although research on osteosarcoma progression has garnered widespread attention, many unknowns remain regarding potential therapeutic targets. PSMD14 [187], a proteasome regulatory subunit and deubiquitinase, is a key oncogenic factor in cancer that regulates various physiological processes and malignant tumor behaviors. Its high expression is associated with sarcoma metastasis prognosis, yet its role in osteosarcoma has not been well studied. Studies indicate that miR-221 and miR-222 [188] are related to tumor metastasis and drug resistance; however, whether they could serve as potential targets in osteosarcoma metastasis and the response to chemotherapy requires further investigation. Additionally, miR-4492 [189], miR-135b-5p [190], and miR-146a-5p [191] have been identified as potential biomarkers for OS metastasis. These are just a few known examples of potential targets in osteosarcoma research. Currently, clinical trials using natural products and miRNAs to treat osteosarcoma are limited. Conducting more clinical trials to verify their efficacy and safety is imperative for advancing clinical applications in this field. Although some breakthroughs have been achieved at this stage, more rigorous and in-depth research is needed to further elucidate the specific clinicopathological significance and potential mechanisms of miRNAs in osteosarcoma. This approach will provide a stronger theoretical foundation and more effective practical guidance for using natural products to treat osteosarcoma, significantly enhancing treatment levels and fostering new developments in this area.

## Figures and Tables

**Table 1:** Therapeutic effects of natural products on osteosarcoma.

Natural Product	Target Gene	Expression Effect	Type of Cell Line	Reference
Psoralidin	FAK PI3K/Akt	Inhibit Osteosarcoma Growth and Metastasis by Downregulating ITGB1 Expression	143B MG63	[31]
Bavachin	STAT3/P53/SLC7A11	Induce Ferroptosis	MG63 HOS	[32]
Cantharidin	miR-214-3p/DKK3	Inactivate β-catenin Nuclear Translocation and LEF1 Translation	U-2OS 143B Saos-2 MG-63 MNNG hFOB1.19	[33]
Baicalein	ERK	Inhibit Cell Development, Metastasis, and EMT and Induce Apoptosis	MG-63	[34]
Naringenin	STAT3-MGST2	Induce Ferroptosis	HOS U2OS MG63	[35]
Piperlongumine	ROS/PI3K/ Akt	Induce Apoptosis and G2/M Phase Arrest	MG63 U2OS	[36]
Metformin	PI3K/AKT/mTOR	Facilitate Osteoblastic Differentiation and M2 Macrophage Polarization	UC-MSC	[37]
Eldecalcitol	PI3K/Akt/mTOR	Accumulate ROS/Induce Apoptosis and Autophagy	MG-63 MC3T3-E1 MLO-Y4	[38]

## Data Availability

Data sharing is not applicable to this article.
